# Supplementary material for: The Role of DNA Topoisomerase Binding Protein 1 (TopBP1) in Genome Stability in Arabidopsis
Source: Plants (Basel). 2021 Nov 24;10(12):2568. doi: 10.3390/plants10122568 (PMC8706423; doi:10.3390/plants10122568)
Supplement: Supplementary file 1 [file plants-10-02568-s001.zip › plants-1459397-supplementary.pdf]

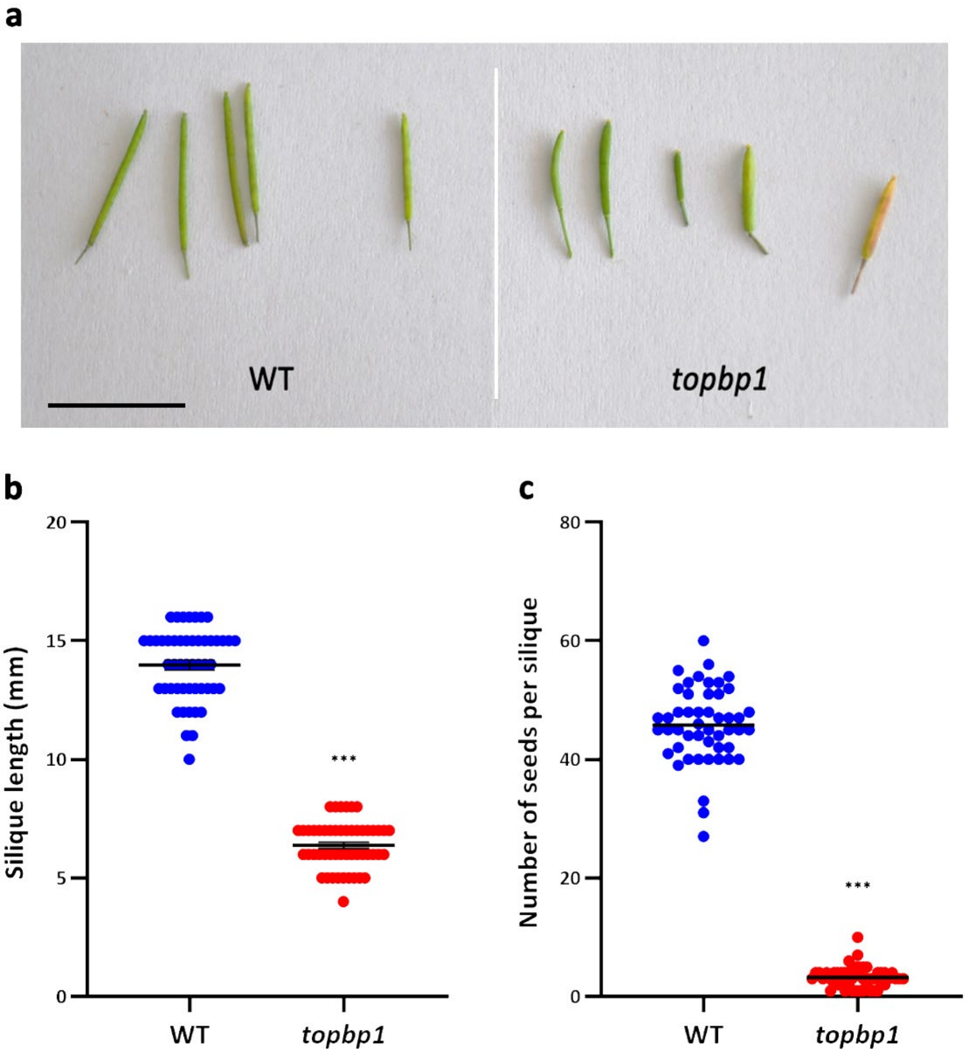

Figure S1. Fertility analysis of WT and *topbp1*. Picture showing representative examples of WT and *topbp1* siliques; scale bar represents 15 mm (**a**). Dot blots represent the silique length (mm) (**b**) and the number of seeds per silique (**c**) in the WT and *topbp1*. Statistical differences between WT and *topbp1* both in silique length and number of seeds per silique were analysed by Welch´s test, *** $p < 0.001$.
